# Supplementary figures and images for: Host biomarkers and combinatorial scores for the detection of serious and invasive bacterial infection in pediatric patients with fever without source
Source: PLoS One. 2023 Nov 13;18(11):e0294032. doi: 10.1371/journal.pone.0294032 (PMC10642781; doi:10.1371/journal.pone.0294032)

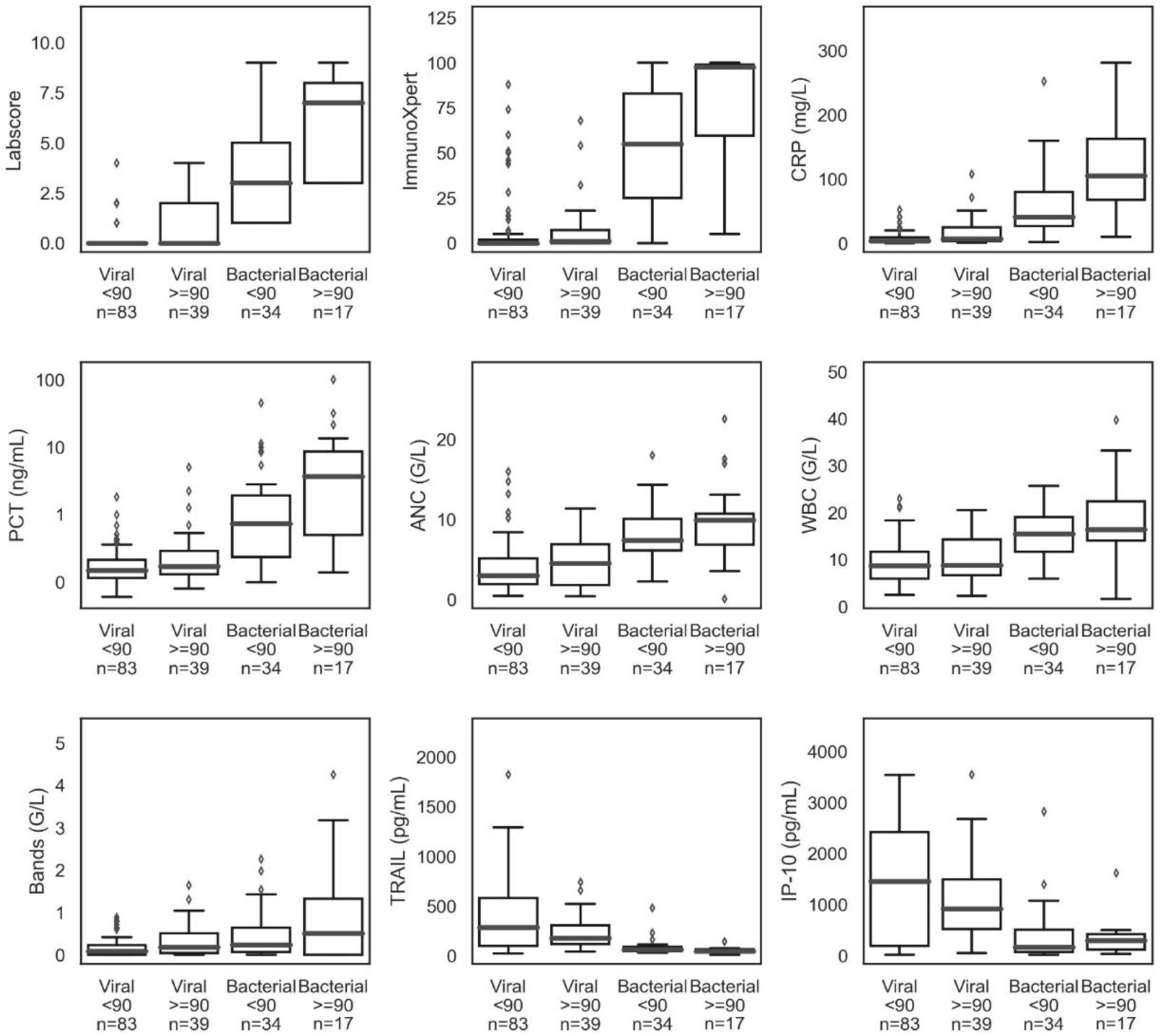

Supplement: S1 Fig — Distribution of biomarker concentrations in patients with FWS according to the final diagnosis (viral or bacterial infection) set by expert panel adjudication (and in control patients for ImmunoXpert and its components) in the subgroup analyzed under the unanimous reference standard (exclusion of children showing non concordant etiology labels assigned by the 3 experts, with an indeterminate etiology or showing equivocal ImmunoXpert score 35 ≤ ImmunoXpert score ≤ 65). (TIF) [file pone.0294032.s001.tif]
