# Supplementary material for: Host biomarkers and combinatorial scores for the detection of serious and invasive bacterial infection in pediatric patients with fever without source
Source: PLoS One. 2023 Nov 13;18(11):e0294032. doi: 10.1371/journal.pone.0294032 (PMC10642781; doi:10.1371/journal.pone.0294032)
Supplement: S2 Table — a The ImmunoXpert is intended for patients ≥ 90 days. The analysis of the ImmunoXpert performance in patients < 90 days is exploratory. (DOCX) [file pone.0294032.s003.docx]

| **Biomarker of interest** | **All patients**  **n=241** | **< 90 days**  **n=155** | **≥ 90 days**  **n=86** | **P value^a^** |
| --- | --- | --- | --- | --- |
| **Labscore** | 0.847 (0.795 to 0.898) | 0.910 (0.864 to 0.957) | 0.805 (0.696 to 0.914) | 0.0826 |
| **ImmunoXpert** | 0.801 (0.730 to 0.871) | 0.810 (0.724 to 0.895) | 0.836 (0.726 to 0.946) | 0.7097 |
| **CRP** | 0.772 (0.696 to 0.848) | 0.823 (0.730 to 0.915) | 0.778 (0.651 to 0.905) | 0.5773 |
| **PCT** | 0.770 (0.694 to 0.846) | 0.809 (0.721 to 0.897) | 0.742 (0.587 to 0.898) | 0.4647 |
| **ANC** | 0.706 (0.625 to 0.788) | 0.759 (0.670 to 0.847) | 0.642 (0.467 to 0.817) | 0.2459 |
| **WBC** | 0.702 (0.619 to 0.785) | 0.738 (0.649 to 0.827) | 0.672 (0.478 to 0.866) | 0.5482 |
| **Bands** | 0.633 (0.529 to 0.738) | 0.646 (0.525 to 0.768) | 0.659 (0.449 to 0.870) | 0.9166 |
| **IP-10** | 0.688 (0.605 to 0.771) | 0.682 (0.580 to 0.784) | 0.706 (0.565 to 0.848) | 0.7857 |
| **TRAIL** | 0.770 (0.697 to 0.843) | 0.762 (0.674 to 0.850) | 0.810 (0.691 to 0.928) | 0.5293 |
