# Supplementary material for: Host biomarkers and combinatorial scores for the detection of serious and invasive bacterial infection in pediatric patients with fever without source
Source: PLoS One. 2023 Nov 13;18(11):e0294032. doi: 10.1371/journal.pone.0294032 (PMC10642781; doi:10.1371/journal.pone.0294032)
Supplement: S4 Table — * Missing data due to insufficient sample. (DOCX) [file pone.0294032.s005.docx]

| **#** | **Age** | **Gender** | **Maximal temperature** | **Labscore** | **Immuno- Xpert** | **CRP** | **PCT** | **ANC** | **WBC** | **Discharge diagnosis** |
| --- | --- | --- | --- | --- | --- | --- | --- | --- | --- | --- |
|  | (days) |  | (°C) |  |  | (mg/L) | (ng/mL) | (G/L) | (G/L) |  |
| **102** | 28 | Female | 38·6 | 4 | 44 | 13 | 5·41 | 7·32 | 12·4 | *Pseudomonas aeruginosa* bacterial meningitis |
| **176** | 97 | Female | 38·2 | 6 | 99 | 49 | 21·3 | 9·93 | 39·7 | *Streptococcus mitis* bacteremia |
| **236** | 308 | Female | 39·6 | 8 | 99 | 154 | 3·69 | 10·74 | 16·4 | *Streptococcus pneumoniae* bacterial meningitis and bacteremia |
| **245** | 41 | Male | 38·4 | 5 | * | 30 | 11·41 | 6·91 | 12·8 | *Escherichia coli* UTI and bacteremia |
| **278** | 90 | Female | 39·7 | 6 | 97 | 82 | 31·8 | 0·08 | 1·6 | *Haemophilus influenzae* meningitis and sepsis |
