# Supplementary material for: Host biomarkers and combinatorial scores for the detection of serious and invasive bacterial infection in pediatric patients with fever without source
Source: PLoS One. 2023 Nov 13;18(11):e0294032. doi: 10.1371/journal.pone.0294032 (PMC10642781; doi:10.1371/journal.pone.0294032)
Supplement: S1 File — (DOCX) [file pone.0294032.s006.docx]

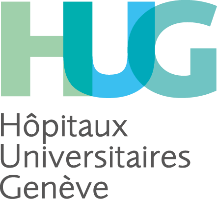
Study Protocol

Original study: Which virus leads to viremia in children with fever without source and do new biomarkers correlate with viral and bacterial infections?

**Part 3: Host biomarkers and combinatorial scores for the detection of serious and invasive bacterial infection in pediatric patients with fever without source**

**National Clinical Trial (NCT) Identifier Number:** NCT03224026

**Principal Investigator: Dr Annick Galetto (annick.galetto@hcuge.ch)**

Funded by: MeMed

Version Number: v.1.0

June 26, 2015

The present version of the protocol only concerns the aspects of correlation between biomarkers and bacterial infections. It is derived from the main study protocol, submitted on June, 26, 2015 to Geneva Cantonal Ethics Committee and accepted (study number **CCER 15-082).**

**ROLES and RESPONSIBILITIES :**


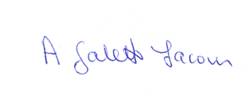


**Principal Investigator:**

Signature: Date: June 26, 2015

Name: Pr Annick Galetto

Title: MD, Deputy Head of Service

Affiliation: Pediatric Emergency Department, Children’s Hospital

Geneva University Hospitals

Av. de la Roseraie 47

CH-1205 Genève

+41 79 55 32 690

[annick.galetto@hcuge.ch](mailto:annick.galetto@hcuge.ch)

**Co-investigators:** Pr Alain Gervaix, MD

Dr Laurence Lacroix, MD

**Sponsor (within the meaning of the Swiss Ordinance on Clinical Trials in Human Research of 20 September 2013, Article 2) :** MeMed, MeMed, Tirat Carmel, Israel

**1. Introduction**

- 1. **Background**

Fever without source (FWS) is defined as a fever 38.0°C/100.4°F and above for less than 7 days with no underlying cause despite a thorough history and physical exam^[[1]](#endnote-1),^^[[2]](#endnote-2)^. Most children below 3 years old presenting with FWS have a self-limited viral infection. However, 10 to 25% of children have an underlying serious bacterial infection (SBI), such as pyelonephritis or pneumonia, or an invasive bacterial infection (IBI), such as meningitis or bacteremia, which can present only as FWS in the early stages^[[3]](#endnote-3)^. Because of lack of specificity in symptoms and signs^[[4]](#endnote-4),^^[[5]](#endnote-5)^, diagnosis is frequently supported by biological exams. Besides blood tests, more invasive laboratory procedures such as lumbar puncture and urinary catheterization are often required. Empirical administration of broad-spectrum antibiotics to prevent SBI cases are often prescribed. Differentiating viral from bacterial infections in children with FWS could avoid invasive investigations and useless antibiotic prescription.

Routinely used microbiological diagnostic tests such as culture, serology, and more recently nucleic acid-based tests (e.g., RT-PCR) can assist the clinician in the etiological determination of the underlying infectious process. However, several challenges remain, including : (i) pathogen detection in cases where the infection site is not readily accessible (e.g., pneumonia), or is unknown (e.g., fever without source); (ii) time to microbiological laboratory results may be lengthy (hours or days); (iii) determination whether a detected bacteria is the disease causative agent or a mere colonizer^[[6]](#endnote-6),^^[[7]](#endnote-7),^^[[8]](#endnote-8)^, and (iv) identification of a virus does not preclude the possibility that an undetected bacteria may be the cause of the underlying illness (i.e. a case of mixed infection with both virus and bacteria)^[[9]](#endnote-9)^.

These challenges can be addressed through monitoring the host's immune response to infection, rather than direct pathogen detection. Proteins that are routinely used to support diagnosis of bacterial infection include procalcitonin (PCT) and C-reactive protein (CRP). However, these markers are sensitive to inter-patient variability, including time from symptom onset, clinical syndrome, and pathogen species^3,^^[[10]](#endnote-10)^. For example, multiple studies found that procalcitonin is valuable for guiding antimicrobial therapy duration and for predicting disease severity^[[11]](#endnote-11),^^[[12]](#endnote-12),^^[[13]](#endnote-13)^. However, its diagnostic accuracy for detecting bacterial etiology in sepsis and pneumonia has been challenged. Elevated CRP levels are suggestive of a bacterial infection^3,^^[[14]](#endnote-14)^, but similar levels may be observed in patients with some viral strains (e.g., adenovirus and influenza)^[[15]](#endnote-15)^, and inflammatory diseases.

Whereas most biomarkers or combinations of biomarkers are responsive to bacterial infections, a new assay (ImmunoXpert) intended for children aged 90 days and more and adults has been recently developed^12,^^[[16]](#endnote-16),^^[[17]](#endnote-17),^^[[18]](#endnote-18)^. This assay measures the concentration of three host-related, serum-based protein biomarkers and combines their levels into a single score that determines the likelihood of bacterial or viral immune response. The score is computed using a predetermined logistic regression formula that was developed and tested on previously collected cohort of >2000 patients. It combines for the first time two viral-induced biomarkers, tumor necrosis factor-related apoptosis-inducing ligand (TRAIL) and interferon γ-induced protein-10 (IP-10), with a bacterial-induced one, C-reactive protein (CRP)^6^. Using predetermined score cut-offs each one of the patients will be classified as having bacterial, viral or equivocal immune response (i.e., inconclusive). It displays three possible outcomes: (1) Viral infection (or non-bacterial aetiology): ImmunoXpert score < 35; (2) Equivocal: 35 ≤ ImmunoXpert score ≤ 65; and (3) Bacterial infection (including mixed bacterial and viral co-infection): ImmunoXpert score > 65.

**1.2 Previous work justifying this trial**

A recent study using the novel ImmunoXpert assay as a single ELISA test, proved to be outstanding in discriminating bacterial from viral infection, without the need to directly sample the infection site and with the ability to differentiate between pathogenic strains of bacteria or viruses and colonizers^[[19]](#endnote-19)^. This signature has already shown high diagnostic accuracy in a heterogeneous study population of febrile inpatients and emergency department arrivals, both children and adults, presenting with diverse clinical syndromes and pathogens^12,^^[[20]](#endnote-20),^^[[21]](#endnote-21),^^[[22]](#endnote-22)^. Rapid (within minutes) and easy to use formats suitable for the point-of-care are under development.

The potential applicability of the TRAIL/IP-10/CRP assay and its comparison with other common biomarkers and the Labscore^3,^^[[23]](#endnote-23),^^[[24]](#endnote-24)^, a biological score based on weighted scoring of procalcitonin (PCT), C-reactive protein (CRP) and urinary dipstick results, has never been studied prospectively in a population of children with FWS. Moreover, limited data were available in the fragile subpopulation of patients aged under 90 days in whom the need is serious for improved diagnostic tools to drive decision-making^[[25]](#endnote-25),^^[[26]](#endnote-26)^. The purpose of this study is to assess the diagnostic accuracy of various individual and combined bacterial- and viral-specific biomarkers, notably the ImmunoXpert and the Labscore, in children and infants under and over 90 days old presenting with FWS.

**2. Methods**

**2.1 Objectives and hypothesis**

To determine the correlation of a combination of biomarkers: CRP with two new viral biomarkers (TNF-related apoptosis-inducing ligand (TRAIL) and Interferon gamma-induced protein-10 (IP-10) for the discrimination between viral or bacterial infections (SBI and IBI).

**Primary objective**

To prospectively investigate the diagnostic accuracy of a host signature (TRAIL, IP-10 and CRP) to reliably predict bacterial infections and to compare its performance to routine individual biomarkers (CRP, PCT, WBC, ANC, TRAIL and IP-10) and to the Labscore. Differential expression of biomarkers and combinatorial scores will be assessed in children with FWS and in control patients for the ImmunoXpert and its components.

**Secondary objectives**

To compare the diagnostic accuracy of the ImmunoXpert, the Labscore, and individual biomarkers to reliably predict IBI.

**2.2 Study design**

Prospective diagnostic accuracy study in infants and children with FWS aged less than 3 years old (consecutive sample).

**2.3 Participants**

**2.3.1 Enrolment**

The study focuses on pediatric patients under 3 years of age presenting to the Pediatric Emergency Department (PED) of Children’s Hospital, Geneva University Hospitals (HUG), with a diagnosis of FWS after a thorough history and physical examination. The physician in charge will describe the purpose and the procedure of the study, possible risks/benefits, as well as rights and responsibilities of participants. If the patient or parents/guardians agree to participate, they will be asked to sign an informed consent form (IC) except if estimated without the competency to understand its content. No analysis will be performed before the signature of the IC.

**2.3.2 Inclusion Criteria**

- Clinical diagnosis of FWS (fever for less than 7 days with no underlying cause determined by history and/or physical exam)
- Age < 3 years old
- Informed consent (IC) given by parent or legal guardian.

**2.3.3 Exclusion Criteria**

- Unavailable blood specimen
- Comorbidities predisposing to infections such as cancer, primary or secondary immunodeficiency, and iatrogenic immunosuppression.

**2.4 Study specimen collection**

After informed consent was obtained, demographic data will be recorded (age, se, as well as delay between fever onset and presentation, and maximum temperature before presentation. Patients will be assessed according to local and international guidelines and recommendations with white blood cell count (WBC), absolute neutrophil count (ANC), band count, CRP, PCT, urinary dipstick and culture, and blood culture. Additional testing (CSF culture, chest X-ray, synovial fluid or stool culture etc.) will remain optional, depending on the clinical evaluation.

An additional 0.6 mL sample will be drawn, to determine CRP, TRAIL and IP-10 values and the ImmunoXpert score. No venous puncture for the additional blood sampling will be performed specifically for the study. Blood sample will be drawn from the venous catheter only in case of venous reflux. No other blood sample nor biological exam will be required for the purpose of the study. No human genetic testing will be performed.

The use of sera from a group of 50 healthy Canadian children from trauma and dental clinics will serve as control patients for TRAIL and IP-10 values, after their use have been approved by Toronto’s Hospital for Sick Children Ethics Committee.

**2.5 Gold standard**

**2.5.1 Expert panel adjudication**

Expert panel adjudication will serve as reference standard. This is a common approach to assigning a final diagnosis in fields where gold standard is lacking, specifically in patients with FWS^[[27]](#endnote-27),^^[[28]](#endnote-28)^. For determining the disease etiology (bacterial or viral), each patient will be assigned a diagnosis set by an independent panel of 3 three senior pediatricians with >10 years of experience, each of which will be blinded to the diagnosis of his/her peers and to the assay results. The experts (one senior pediatric infectious-disease specialist and two senior pediatric emergency physicians) will independently perform an initial etiologic classification (viral, bacterial or indeterminate etiology). Then, they will be asked to independently classify patients with indeterminate diagnosis into suspected viral or bacterial infection, as recommended in most studies using this kind of reference standard. The combined results of the experts will permit to create the reference standard as a “majority” diagnosis: cases will be included under the etiology label assigned by minimum 2 of the 3 experts.

Other definitions of reference standard will also be investigated to assess the robustness of the findings: microbiological diagnosis and unanimous reference standard.

**2.5.2 Microbiological diagnosis**

A microbiological diagnosis will be established using below-mentioned predefined criteria to classify patients as follows: (1) bacteriologically proven SBI or IBI or (2) no proof of bacterial infection.

**Definition of SBI**

Microbiological serious bacterial infection is defined by isolation of a bacterial pathogen from any urine, synovial fluid, bone, or stool specimen. Microbiological urinary tract infection (UTI) will be defined according to the AAP Subcommittee on Urinary Tract Infection and Steering Committee on Quality Improvement and Management, based on a urine specimen obtained through urinary tract catheterization or clean catch of mid-stream urine^[[29]](#endnote-29)^:

- Patients > 60 days old: abnormal urinalysis defined by the presence of positive leukocyte esterase, nitrite or pyuria (>5 white blood cells (WBCs) per high-power field) and culture growth of at least 50’000 colony-forming units (cfu) per mL of a uropathogen
- Patients ≤ 60 days old: growth of 50’000 cfu/mL or more of a uropathogen, or growth of 10’000 cfu/mL or more of a single uropathogen in association with an abnormal urinalysis.

**Definition of IBI**

Invasive bacterial infection (IBI) is defined as isolation of a compatible bacterial pathogen in blood or cerebrospinal fluid culture.

**2.5.3 Unanimous reference standard**

Analyses will also be performed under a “unanimous” reference standard, whereby cases will be included in the analysis only if the same etiology label was assigned by all 3 experts, after exclusion of indeterminate cases.

**2.6 Methods of measurement and data collection**

Biological exams will be performed in the Laboratory Department at Geneva University Hospitals. Demographic and clinical data will be collected on case report forms (CRFs), and then entered on a secure institutional database.

**2.7 Power and sample size calculation**

The sample size of this ancillary study is driven by the main study on viremia in patients with FWS, in which a sample of 50 viremic patients is planned[^20^](#_ENREF_20). To reach this target, assuming a 10-20% prevalence of viremia, the enrolment of 400 patients is anticipated, but the enrolment of patients will terminate when the number of patients with viremia will corresponded to the target. A power calculation for this ancillary study is conducted to determine the detectable difference in area under the ROC curve (of any biomarker compared with the ImmunoXpert) with a power of 80% under various sample sizes (0.08 detectable difference with a sample size of 100 to 0.10 with a sample size of 264 patients). The power to detect a difference in areas under ROC curve should be acceptable for the evaluation of biomarker accuracy.

Concerning power calculation, we assume (1) a two-sided risk alpha of 0.05, (2) a correlation between ImmunoXpert and the other marker of 0.5 both in patients with a bacterial infection and in those with a viral infection and (3) a viral infection rate 3 times higher than that of bacterial infection. Using the method of sample size calculation for comparison of paired binormal ROC curves proposed by Obuchowski et al^[[30]](#endnote-30)^, the needed sample size is 264 patients (66 with a bacterial infection and 198 with a viral infection).

**2.8 Blinding**

Experts will access to full medical records but will remain blinded to the diagnosis of their peers and to TRAIL, IP-10 and ImmunoXpert results; CRP, PCT and urinary dipstick data will be available to the experts to determine the etiology label. The ImmunoXpert test will be performed on anonymized samples. The index test and panel expert reference standard outcomes will be locked prior to unblinding.

**2.9 Confidentiality**

Relevant patient data will be recorded on individual case report forms (CRFs) under an individual identifying code assigned to each participant that does not provide any identifying information. Data will be entered on the day of enrolment and after definitive laboratory results by study investigators. The information contained within the CRFs will be transferred to a computerized database and will be exclusively accessible to the study team. Data will be encoded with the date of birth and stored for 10 years.

**2.10 Statistical analysis plan**

Biomarkers will be described in patients with bacterial and viral infections by median and interquartile intervals. Mann-Whitney test will be used to compare these groups. Diagnostic performances of biological scores and markers in identifying bacterial infections will be assessed by non-parametric ROC curves. The areas under the ROC curves will be assessed and compared using the non-parametric approach proposed by Delong et al^[[31]](#endnote-31)^. This approach accounts for paired data when two compared markers are measured in the same person. Sensitivities and specificities will be reported with the Clopper-Pearson exact 95% confidence intervals. The two-sided risk alpha will be 0.05 in all comparisons. Software used for statistical analyses will be R version 3.5.2 (R Core Team (2018). R: A language and environment for statistical computing. R Foundation for Statistical Computing, Vienna, Austria. URL <https://www.R-project.org/>).

No interim analyses will be performed as the implication for the patient is short in duration and doesn’t show any serious potential outcome.

**3. Financial issues**

The work-up dedicated to routine standard of care will be borne by the patient’s insurance. Costs dedicated to enrolment, database maintenance and statistics will be covered by research funds. The additional testing (ImmunoXpert score determination) performed for the purpose of the study will be processed and funded by MeMed.

**4. Risk and benefits for the patient**

No patient will have a blood puncture specifically for the study as only patients requiring blood testing for the usual clinical workup will be included. Therefore, there will be no additional harm related to blood sampling. The extra amount of blood required for the study (0.6 mL) is negligible, even in infants since it represents less than 0.5% of the total amount of circulating blood volume.

There is no direct benefit for the patient. However, we hope this study will improve the care of children presenting with FWS through better discrimination between viral and bacterial infections, avoiding unnecessary diagnostic procedures and antibiotic treatment.

**5. Criteria for discontinuing study participation**

Any parent or caregiver willing to interrupt the child’s participation to the present study is free. The following medical care won’t be influenced by any decision to discontinue the study.

**6. Adherence**

Due to our experience gathered from previous studies, we anticipate a very low rate of refusals or drop-outs since there is no other intervention than drawing an additional 0.6 mL blood sample at the time of the routine standard lab workup. Moreover, there is no need for follow up.

**7. Study schedule and milestones**

Patients will be enrolled in the study immediately after approval from the Ethics Committee (CCER) and will be conducted for a minimum of 4 years, which is the estimated time to recruit 400 patients in order to have enough viremic patients (VirEF study part 1).

| Study year 1 | Study year 2 | Study year 3 | Study year 4 | Close-out |
| --- | --- | --- | --- | --- |
| Patient enrolment | Patient enrolment | Patient enrolment | Patient enrolment | Expert panel review of cases |
|  |  |  |  | Data analysis |
|  |  |  |  | Preparation for publication |

Table 1. Timeframe of the study

**8. References**

1. Esposito S, Rinaldi VE, Argentiero A, Farinelli E, Cofini M, D'Alonzo R, Mencacci A, Principi N. Approach to Neonates and Young Infants with Fever without a Source Who Are at Risk for Severe Bacterial Infection. Mediators Inflamm. 2018 Nov 26;2018:4869329. doi: 10.1155/2018/4869329. PMID: 30581369; PMCID: PMC6287153. [↑](#endnote-ref-1)
2. Wing R, Dor MR, McQuilkin PA. Fever in the pediatric patient. Emerg Med Clin North Am. 2013 Nov;31(4):1073-96. doi: 10.1016/j.emc.2013.07.006. Epub 2013 Sep 26. PMID: 24176480. [↑](#endnote-ref-2)
3. Galetto-Lacour A, Zamora SA, Andreola B, Bressan S, Lacroix L, Da Dalt L, Gervaix A. Validation of a laboratory risk index score for the identification of severe bacterial infection in children with fever without source. Arch Dis Child. 2010 Dec;95(12):968-73. doi: 10.1136/adc.2009.176800. Epub 2010 Jun 1. PMID: 20515973. [↑](#endnote-ref-3)
4. Ishimine P. Risk stratification and management of the febrile young child. Emerg Med Clin North Am. 2013 Aug;31(3):601-26. doi: 10.1016/j.emc.2013.05.003. Epub 2013 Jul 5. PMID: 23915596. [↑](#endnote-ref-4)
5. Baraff LJ. Management of infants and young children with fever without source. Pediatr Ann. 2008 Oct;37(10):673-9. doi: 10.3928/00904481-20081001-01. PMID: 18972849. [↑](#endnote-ref-5)
6. Caserta MT, Hall CB, Schnabel K, Lofthus G, Marino A, Shelley L, Yoo C, Carnahan J, Anderson L, Wang H. Diagnostic assays for active infection with human herpesvirus 6 (HHV-6). J Clin Virol. 2010 May;48(1):55-7. doi: 10.1016/j.jcv.2010.02.007. Epub 2010 Mar 7. PMID: 20211581; PMCID: PMC2855742. [↑](#endnote-ref-6)
7. Flamand L, Komaroff AL, Arbuckle JH, Medveczky PG, Ablashi DV. Review, part 1: Human herpesvirus-6-basic biology, diagnostic testing, and antiviral efficacy. J Med Virol. 2010 Sep;82(9):1560-8. doi: 10.1002/jmv.21839. PMID: 20648610. [↑](#endnote-ref-7)
8. Ambrosioni J, Bridevaux PO, Wagner G, Mamin A, Kaiser L. Epidemiology of viral respiratory infections in a tertiary care centre in the era of molecular diagnosis, Geneva, Switzerland, 2011-2012. Clin Microbiol Infect. 2014 Sep;20(9):O578-84. doi: 10.1111/1469-0691.12525. Epub 2014 Jan 24. PMID: 24382326; PMCID: PMC7128668. [↑](#endnote-ref-8)
9. Cabrerizo M, Calvo C, Rabella N, Muñoz-Almagro C, del Amo E, Pérez-Ruiz M, Sanbonmatsu-Gámez S, Moreno-Docón A, Otero A, Trallero G; study group of Enterovirus and parechovirus infections in children under 3 years-old, Spain (PI12-00904). Design and validation of a real-time RT-PCR for the simultaneous detection of enteroviruses and parechoviruses in clinical samples. J Virol Methods. 2014 Nov;208:125-8. doi: 10.1016/j.jviromet.2014.08.008. Epub 2014 Aug 22. PMID: 25152526. [↑](#endnote-ref-9)
10. Manzano S, Bailey B, Gervaix A, Cousineau J, Delvin E, Girodias JB. Markers for bacterial infection in children with fever without source. Arch Dis Child. 2011 May;96(5):440-6. doi: 10.1136/adc.2010.203760. Epub 2011 Jan 29. PMID: 21278424. [↑](#endnote-ref-10)
11. Nijman RG, Moll HA, Smit FJ, Gervaix A, Weerkamp F, Vergouwe Y, de Rijke YB, Oostenbrink R. C-reactive protein, procalcitonin and the lab-score for detecting serious bacterial infections in febrile children at the emergency department: a prospective observational study. Pediatr Infect Dis J. 2014 Nov;33(11):e273-9. doi: 10.1097/INF.0000000000000466. PMID: 25093971. [↑](#endnote-ref-11)
12. Oved K, Cohen A, Boico O, Navon R, Friedman T, Etshtein L, Kriger O, Bamberger E, Fonar Y, Yacobov R, Wolchinsky R, Denkberg G, Dotan Y, Hochberg A, Reiter Y, Grupper M, Srugo I, Feigin P, Gorfine M, Chistyakov I, Dagan R, Klein A, Potasman I, Eden E. A novel host-proteome signature for distinguishing between acute bacterial and viral infections. PLoS One. 2015 Mar 18;10(3):e0120012. doi: 10.1371/journal.pone.0120012. PMID: 25785720; PMCID: PMC4364938. [↑](#endnote-ref-12)
13. Bonner AB, Monroe KW, Talley LI, Klasner AE, Kimberlin DW. Impact of the rapid diagnosis of influenza on physician decision-making and patient management in the pediatric emergency department: results of a randomized, prospective, controlled trial. Pediatrics. 2003 Aug;112(2):363-7. doi: 10.1542/peds.112.2.363. PMID: 12897288. [↑](#endnote-ref-13)
14. Petty TJ, Cordey S, Padioleau I, Docquier M, Turin L, Preynat-Seauve O, Zdobnov EM, Kaiser L. Comprehensive human virus screening using high-throughput sequencing with a user-friendly representation of bioinformatics analysis: a pilot study. J Clin Microbiol. 2014 Sep;52(9):3351-61. doi: 10.1128/JCM.01389-14. Epub 2014 Jul 9. PMID: 25009045; PMCID: PMC4313162. [↑](#endnote-ref-14)
15. Popowitch EB, O'Neill SS, Miller MB. Comparison of the Biofire FilmArray RP, Genmark eSensor RVP, Luminex xTAG RVPv1, and Luminex xTAG RVP fast multiplex assays for detection of respiratory viruses. J Clin Microbiol. 2013 May;51(5):1528-33. doi: 10.1128/JCM.03368-12. Epub 2013 Mar 13. PMID: 23486707; PMCID: PMC3647947. [↑](#endnote-ref-15)
16. Eden E, Srugo I, Gottlieb T, Navon R, Boico O, Cohen A, Bamberger E, Klein A, Oved K. Diagnostic accuracy of a TRAIL, IP-10 and CRP combination for discriminating bacterial and viral etiologies at the Emergency Department. J Infect. 2016 Aug;73(2):177-80. doi: 10.1016/j.jinf.2016.05.002. Epub 2016 May 30. PMID: 27255416. [↑](#endnote-ref-16)
17. Srugo I, Klein A, Stein M, Golan-Shany O, Kerem N, Chistyakov I, Genizi J, Glazer O, Yaniv L, German A, Miron D, Shachor-Meyouhas Y, Bamberger E, Oved K, Gottlieb TM, Navon R, Paz M, Etshtein L, Boico O, Kronenfeld G, Eden E, Cohen R, Chappuy H, Angoulvant F, Lacroix L, Gervaix A. Validation of a Novel Assay to Distinguish Bacterial and Viral Infections. Pediatrics. 2017 Oct;140(4):e20163453. doi: 10.1542/peds.2016-3453. Epub 2017 Sep 13. PMID: 28904072. [↑](#endnote-ref-17)
18. Ashkenazi-Hoffnung L, Oved K, Navon R, et al. A host-protein signature is superior to other biomarkers for differentiating between bacterial and viral disease in patients with respiratory infection and fever without source: a prospective observational study. Eur J Clin Microbiol Infect Dis. 2018;37:1361-1371. [↑](#endnote-ref-18)
19. Feigin RD, C.J., Adenoviruses, in Textbook of pediatric infectious diseases. 2004, WB Saunders: Philadelphia. p. 1843–1856. [↑](#endnote-ref-19)
20. Eden E, Srugo I, Gottlieb T, Navon R, Boico O, Cohen A, Bamberger E, Klein A, Oved K. Diagnostic accuracy of a TRAIL, IP-10 and CRP combination for discriminating bacterial and viral etiologies at the Emergency Department. J Infect. 2016 Aug;73(2):177-80. doi: 10.1016/j.jinf.2016.05.002. Epub 2016 May 30. PMID: 27255416. [↑](#endnote-ref-20)
21. Srugo I, Klein A, Stein M, Golan-Shany O, Kerem N, Chistyakov I, Genizi J, Glazer O, Yaniv L, German A, Miron D, Shachor-Meyouhas Y, Bamberger E, Oved K, Gottlieb TM, Navon R, Paz M, Etshtein L, Boico O, Kronenfeld G, Eden E, Cohen R, Chappuy H, Angoulvant F, Lacroix L, Gervaix A. Validation of a Novel Assay to Distinguish Bacterial and Viral Infections. Pediatrics. 2017 Oct;140(4):e20163453. doi: 10.1542/peds.2016-3453. Epub 2017 Sep 13. PMID: 28904072. [↑](#endnote-ref-21)
22. Ashkenazi-Hoffnung L, Oved K, Navon R, et al. A host-protein signature is superior to other biomarkers for differentiating between bacterial and viral disease in patients with respiratory infection and fever without source: a prospective observational study. Eur J Clin Microbiol Infect Dis. 2018;37:1361-1371. [↑](#endnote-ref-22)
23. Lacour AG, Zamora SA, Gervaix A. A score identifying serious bacterial infections in children with fever without source. Pediatr Infect Dis J. 2008 Jul;27(7):654-6. doi: 10.1097/INF.0b013e318168d2b4. PMID: 18536624. [↑](#endnote-ref-23)
24. Lacroix L, Manzano S, Vandertuin L, Hugon F, Galetto-Lacour A, Gervaix A. Impact of the lab-score on antibiotic prescription rate in children with fever without source: a randomized controlled trial. PLoS One. 2014 Dec 11;9(12):e115061. doi: 10.1371/journal.pone.0115061. PMID: 25503770; PMCID: PMC4263728. [↑](#endnote-ref-24)
25. Kimberlin DW, Poole CL. Assessing the Febrile Child for Serious Infection: A Step Closer to Meaningful Rapid Results. Pediatrics. 2017 Oct;140(4):e20171210. doi: 10.1542/peds.2017-1210. Epub 2017 Sep 13. PMID: 28904071. [↑](#endnote-ref-25)
26. van Houten CB, de Groot JAH, Klein A, Srugo I, Chistyakov I, de Waal W, Meijssen CB, Avis W, Wolfs TFW, Shachor-Meyouhas Y, Stein M, Sanders EAM, Bont LJ. A host-protein based assay to differentiate between bacterial and viral infections in preschool children (OPPORTUNITY): a double-blind, multicentre, validation study. Lancet Infect Dis. 2017 Apr;17(4):431-440. doi: 10.1016/S1473-3099(16)30519-9. Epub 2016 Dec 22. PMID: 28012942. [↑](#endnote-ref-26)
27. Bertens LC, Broekhuizen BD, Naaktgeboren CA, Rutten FH, Hoes AW, van Mourik Y, Moons KG, Reitsma JB. Use of expert panels to define the reference standard in diagnostic research: a systematic review of published methods and reporting. PLoS Med. 2013 Oct;10(10):e1001531. doi: 10.1371/journal.pmed.1001531. Epub 2013 Oct 15. PMID: 24143138; PMCID: PMC3797139. [↑](#endnote-ref-27)
28. van Houten CB, Naaktgeboren CA, Ashkenazi-Hoffnung L, Ashkenazi S, Avis W, Chistyakov I, Corigliano T, Galetto A, Gangoiti I, Gervaix A, Glikman D, Ivaskeviciene I, Kuperman AA, Lacroix L, Loeffen Y, Luterbacher F, Meijssen CB, Mintegi S, Nasrallah B, Papan C, van Rossum AMC, Rudolph H, Stein M, Tal R, Tenenbaum T, Usonis V, de Waal W, Weichert S, Wildenbeest JG, de Winter-de Groot KM, Wolfs TFW, Mastboim N, Gottlieb TM, Cohen A, Oved K, Eden E, Feigin PD, Shani L, Bont LJ; IMPRIND consortium. Expert panel diagnosis demonstrated high reproducibility as reference standard in infectious diseases. J Clin Epidemiol. 2019 Aug;112:20-27. doi: 10.1016/j.jclinepi.2019.03.010. Epub 2019 Mar 28. PMID: 30930247. [↑](#endnote-ref-28)
29. Subcommittee on Urinary Tract Infection, Steering Committee on Quality Improvement and Management; Roberts KB. Urinary tract infection: clinical practice guideline for the diagnosis and management of the initial UTI in febrile infants and children 2 to 24 months. Pediatrics. 2011 Sep;128(3):595-610. doi: 10.1542/peds.2011-1330. Epub 2011 Aug 28. PMID: 21873693. [↑](#endnote-ref-29)
30. Xiao-Hua Zhou NAO, Donna K. McClish. Statistical Methods in Diagnostic Medicine. 2nd ed2011. [↑](#endnote-ref-30)
31. DeLong ER, DeLong DM, Clarke-Pearson DL. Comparing the areas under two or more correlated receiver operating characteristic curves: a nonparametric approach. Biometrics. 1988 Sep;44(3):837-45. PMID: 3203132. [↑](#endnote-ref-31)
