# Supplementary material for: Host biomarkers and combinatorial scores for the detection of serious and invasive bacterial infection in pediatric patients with fever without source
Source: PLoS One. 2023 Nov 13;18(11):e0294032. doi: 10.1371/journal.pone.0294032 (PMC10642781; doi:10.1371/journal.pone.0294032)
Supplement: Attachment — Submitted filename: Response to Reviewers_PONE_D-23-17053.docx [file pone.0294032.s007.docx]

Département de la femme, de l’enfant et de l’adolescent

Service d’Accueil et d’Urgences Pédiatriques

### Pr Alain GERVAIX

### Chef de service

🕾	+41 22 372 45 41

🕾	+41 79 553 24 10

Fax	+41 22 372 54 23

- [alain.gervaix@hcuge.ch](mailto:alain.gervaix@hcuge.ch)

**Pre Annick GALETTO**

Médecin adjointe agrégée

- +41 22 372 46 45

🕾	+41 79 553 26 90

- [annick.galetto@hcuge.ch](mailto:annick.galetto@hcuge.ch)

**Pr Sergio MANZANO**

Médecin adjoint agrégé

- +41 22 372 45 75

🕾	+41 79 553 26 18

- [sergio.manzano@hcuge.ch](mailto:sergio.manzano@hcuge.ch)

**Dre Laurence LACROIX-DUCARDONNOY**

Médecin adjointe agrégée

- +41 22 372 50 82

🕾	+41 79 553 25 81

- [laurence.lacroix@hcuge.ch](mailto:laurence.lacroix@hcuge.ch)

**Dr Michael BUYCK**

Médecin adjoint

🕾	+41 79 553 18 78

- [michael.buyck@hcuge.ch](mailto:michael.buyck@hcuge.ch)

**Dre Marine GRANGE-MERCIER**

Médecin adjoint

🕾	+41 79 553 97 83

- [marine.mercier@hcuge.ch](mailto:marine.mercier@hcuge.ch)

**Dr Cyril SAHYOUN**

Médecin adjoint

🕾	+41 79 553 97 81

- [cyril.sahyoun@hcuge.ch](mailto:cyril.sahyoun@hcuge.ch)

**Dr Johan SIEBERT**

Médecin adjoint

🕾	+41 79 553 40 72

- [johan.siebert@hcuge.ch](mailto:johan.siebert@hcuge.ch)

**M. Kevin HADDAD**

Infirmier responsable d’équipe de soins

🕾	+41 79 553 31 63

- [kevin.haddad@hcuge.ch](mailto:kevin.haddad@hcuge.ch)

**Mme Virginie LACOFFRETTE**

Infirmière responsable d’équipe de soins

🕾	+41 79 553 31 66

- [virginie.lacoffrette@hcuge.ch](mailto:virginie.lacoffrette@hcuge.ch)

**Mme Aude ANGIOLILLI**

Secrétaire

🕾	+41 22 372 45 97

- [aude.angiolilli@hcuge.ch](mailto:aude.angiolilli@hcuge.ch)

**Dr Novel N. Chegou**

**Academic Editor**

**PLOS ONE**

Geneva, October 11, 2023

**Concerns: Revised manuscript PONE-D-23-17053 (Host biomarkers and combinatorial scores for the detection of serious and invasive bacterial infection in pediatric patients with fever without source)**

Dear Editor,

We are grateful for the revision of our manuscript entitled: “Host biomarkers and combinatorial scores for the detection of serious and invasive bacterial infection in pediatric patients with fever without source” and for your comments and suggestions. You will find below a detailed point by point response to each point raised during the review process to correspond to the journal requirements and to address the comments of both the academic editor and the reviewers.

**Response to the Academic Editor and Journal requirements:**

**1.** **PLOS ONE's style requirements**: our manuscript has been checked so that it meets PLOS ONE's style requirements, including those for file naming.

**2. Funding of the study**: there was no additional external funding other than those already mentioned in the original funding statement. As you suggested, our Funding Statement should be updated as follows:

“This was an investigator-driven study. Alain Gervaix received financial support from MeMed to support the study. The funder provided support in the form of salaries for authors NM, LE, LS, ES, EB, RN, TG, KO, EE. The specific roles of these authors are articulated in the ‘author contributions’ section. The funders and study sponsors had no role in the design and conduct of the study, and in interpretation of clinical data. The first version of the manuscript was written by Laurence Lacroix. The funder (MeMed)

participated in data collection and analysis (ImmunoXpert assays) and in reviewing the manuscript. Annick Galetto-Lacour and Arnaud L'Huillier received funding to support the study from the Gertrude von Meissner Foundation, the Ernst and Lucie Schmidheiny Foundation and the University Hospitals of Geneva's Research and Development Project Grant. There was no additional external funding received for this study.” We would be grateful if you change the online submission form on our behalf.

**3**. **Competing Interests Statement.** Thank you for your comment on author affiliation. We had previously mentioned that some of the authors are employees of MeMed, one of the funding organizations. Our statements relating to the author contributions have been revised in the Author Contributions section of the online submission form. The amended statement of competing interests now explicitly states: “The financial support received by AG from MeMed as well as the commercial affiliation of authors NM, LE, LS, ES, EB, RN, TG, KO, EE with MeMed does not alter our adherence to all PLOS ONE policies on sharing data and materials. Other authors had no relationship relating to employment, consultancy, patents, products in development, marketed products, etc. with any of the funding organizations.”

**4 & 5.** **Data Availability statement.** We had previously stated that data were available “upon request”. We now confirm that all data underlying the findings in our study will be freely available from a public repository (Dryad) as soon as the manuscript is accepted for publication (<https://doi.org/10.5061/dryad.gxd2547s8>). This information has been updated in our Data Availability statement. However, as suggested, before before uploading the data and abstract and making them public, we will wait for your confirmation that the manuscript is accepted for publication.

**6. Author affiliation.** The corresponding author (LL) is affiliated with the institute chosen for direct billing option (University of Geneva, Geneva, Switzerland) (page 1 lines 6 and 13).

**7.** The term “data not shown” was not exact. We were meaning “results not shown in the main manuscript file”. However, this assertion refers to an already mentioned table in the supporting information file (S2 Table) that shows all data underlying this comment. The phrase “data not shown” has been replaced by the indication for the corresponding table in the supporting information file (S2 Table) (page 12, line 280).

**8. Ethics statement.** The full name of ethics committee (Cantonal Committee on Ethics in Scientific Research of Geneva, CCER) who approved our study has been added (page 6 line 146). The need for obtaining written informed consent before inclusion has been added in the revised version of the manuscript (page 6 line 148).

**9. Reference list**. The reference list has been updated to address the comments from the Editor (details on the Labscore, ref 7-8) and from Reviewer # 1, see below (ref 13-14).

**Response to the Reviewers:**

- **Comment from the Reviewer**: For readers who may not be familiar with the labscore, I encourage the authors to explain in more detail how the labscore is derived, what cutoffs it has etc.

**Author response**: Thank you for your suggestion. Details on the Labscore (derivation and validation of the score), and on the cutoffs and recommendations for use have been added in the revised version of the manuscript (page 3-4 line 70-81).

- **Comment from the Reviewer**: Please explain why Canadian children were used as healthy controls for a study on Swiss patients.

**Author response**: Thank you for your comment. One of the goals in our study was the determination of differential biomarker expression between children with fever without source and healthy children. Facing the ethical challenge of performing blood puncture in healthy control children with no fever and no current infection, we decided to use preexisting blood samples to test the relevant biomarkers. We did not find any existing healthy pediatric control samples in Switzerland, but we found an existing healthy Canadian cohort to address this precise objective. This has been added to the revised version of the manuscript (page 6 line 137-143).

- **Comment from the Reviewer**: Line 247: The data for the unanimous reference standard subgroup should be shown in the supplement.

**Author response**: As mentioned above in the response to the academic editor comments, the term “data not shown” referred to a table not shown in the main manuscript file (but that was already included in the supplemental online materiel as S2 Table). The phrase “data not shown” has been replaced by the corresponding table in the supporting information file (S2 Table) (page 12, line 280).

- **Comment from the Reviewer** Explain the asterisk in Table S4 for patient number 245 in the column ImmunoXpert.

**Author response**: Thank you for this detail that we had missed. The asterisk in Table S4 in the ImmunoXpert column of patient 245 refers to insufficient blood sample to perform the test. This has been added in the Supporting Information section of the revised manuscript (page 22 line 553).

- **Comment from the Reviewer**: The false negatives are problematic. The authors should elaborate on the failure of the test in these cases, which were bacteremia or meningitis cases, and also discuss and contextualize with previous studies and their false negatives.

**Author response**: Thank you for your suggestion concerning false negatives cases that are indeed problematic. We had previously elaborated on patients with **invasive** bacterial infections (IBI). All five of them, included bacteremia or meningitis cases mentioned above, would have been detected using an ImmunoXpert ≥ 35, a Labscore threshold of 1 or 3, or PCT ≥ 0.5 ng/mL. However, WBC ≥ 15 G/L or CRP ≥ 40 mg/L would have detected respectively only two and three of these infections (page 15 line 333-341). False negative cases for the ImmunoXpert were patients with a low ImmunoXpert score (<35) but classified as bacterial infections by the panel of experts. As stated in the text (page 15 line 322-327), these cases were mainly patients with a final diagnosis of fever without source, or urinary tract infections. In our study, no DMSA scan was performed to differentiate UTI with or without kidney involvement. Patients with UTI diagnosis and low (<35) ImmunoXpert scores could represent the proportion of patients with cystitis, which is not a serious bacterial infection, but which can present with low grade fever in up to 30% of cases according to the literature, as it was already stated in the manuscript (page 18 line 394-397). The ImmunoXpert assay has been derived from a population of febrile children aged more than 3 months old and adults with various underlying etiologies. It has never been tested specifically in the subgroup of pediatric patients suffering from FWS and data in children less than 3 months were also limited in patients with a definite infection. It is somewhat difficult to contextualize results on the ImmunoXpert in pediatric patients with FWS with the existing literature since this is the first study to assess the assay in this context. This has been added in the discussion section (page 17 line 373-379).

- **Comment from the Reviewer**: The references seem in part to be older, in light of newer studies on the studied marker, the authors should cite and discuss findings from other studies, e.g. this systematic review: PMID 34015531; a large study on a broad pediatric cohort: PMID 34768022; and a very recent study on older patients: PMID 37270059.

**Author response**: Thank you for the suggested references. The systematic review that is mentioned focuses on biomarkers in both adult and pediatric patients with respiratory tract infection, which is not the scope of our study, but it has now been cited as a reference (ref 14, PMID 34015531). The study on the TRAIL/IP-10/CRP signature in a broad pediatric cohort refers to children > 90 days of age with either respiratory tract infections or fever without source. It has been cited as a reference in the introduction section (ref 13, PMID 34768022). The third reference mentioned (PMID 37270059) has not been cited since it refers to febrile adult patients only (median age 56 years old) with suspected LRTI (and not with fever without source).

We are grateful to the Reviewers and the Editor for the time spent in revising our manuscript and for their precious input and for considering this article for publication.

Sincerely,

Dr Laurence Lacroix-Ducardonnoy, MD

Pediatric Emergency Department

Children’s Hospital, University Hospitals of Geneva and Geneva University
